# A Neighbourhood-oriented approach to foster healthy ageing in low socioeconomic older adults: development and protocol for evaluation through intervention mapping

**DOI:** 10.1093/her/cyae041

**Published:** 2024-12-14

**Authors:** Lieke J E Duijsens, Lilian Lechner, Denise A Peels, Catherine A W Bolman

**Affiliations:** Department of Health Psychology, Faculty of Psychology, Open Universiteit, Valkenburgerweg 177, 6401 DL Heerlen, The Netherlands; Department of Health Psychology, Faculty of Psychology, Open Universiteit, Valkenburgerweg 177, 6401 DL Heerlen, The Netherlands; Department of Health Psychology, Faculty of Psychology, Open Universiteit, Valkenburgerweg 177, 6401 DL Heerlen, The Netherlands; Department of Health Psychology, Faculty of Psychology, Open Universiteit, Valkenburgerweg 177, 6401 DL Heerlen, The Netherlands

## Abstract

The rapid ageing of our society poses significant challenges, including an increase in chronic diseases and loneliness among older adults, leading to higher demands for care and support. Addressing these needs requires an integral approach, especially among older adults with low socioeconomic status (SES). This article details the development of the Neighbourhood Active & Connected (NAC) intervention, using the Intervention Mapping framework to expand an evidence-based physical activity intervention into a neighbourhood-oriented, holistic and systemic strategy for healthy ageing. NAC focuses on enhancing physical activity, social connectedness, and digital literacy. It was developed through co-creation with local stakeholders and low-SES older adults, ensuring optimal alignment with their needs and capabilities. Performance and change objectives were established based on literature consultation and an extensive need assessment, with intervention materials refined through engagement with the target group. The results of a large-scale cluster randomised controlled trial will be utilised to evaluate NAC’s impact on physical activity, loneliness, social cohesion, health-related quality of life, and digital literacy among low-SES community-dwelling older adults. This paper aims to provide a detailed roadmap for researchers and practitioners to adapt, implement and evaluate similar interventions, promoting healthy ageing in low socio-economic contexts.

## Introduction

The world population is ageing rapidly, with older adults making up a growing proportion of society [1]. In the Netherlands, the number of adults aged 65 years and older is expected to grow from 3.5 million in 2021 to 4.8 million by 2040, representing nearly 23% of its total population [2]. With ageing comes an increase in the prevalence of chronic diseases [3] and loneliness among older adults [4], which has implications for health systems, social policies and economic sustainability. These societal effects of ageing might be even more pronounced among communities with a low socioeconomic status (SES), as a low SES negatively impacts both physical [5, 6] and psychological [7] well-being. Furthermore, a low SES is associated with a decreased life expectancy [8], particularly in older age [9], and a lower oral health-related quality of life [10]. As a result, there is significant pressure on both formal and informal care systems to support older adults, especially those with a low SES. Hence, it is crucial that older adults maintain their independence and vitality for as long as possible.

There is a growing emphasis on *healthy ageing* to address these challenges, which goes beyond merely maintaining good health by focusing on the quality of life, functionality and overall well-being as individuals grow older [11, 12]. Supporting this concept, research suggests that healthy ageing in older adults should be approached holistically, given its multifaceted nature and its interconnectedness with factors that affect overall well-being. A *holistic* or *integral approach* integrates various life domains by connecting biological, psychological, behavioural, social and environmental determinants [13]. It also emphasises the importance of considering older adults as a whole, focusing on their health conditions and their capabilities, support networks and societal backgrounds [14].

The need for an integral approach to support healthy ageing is particularly urgent for low-SES older adults. This approach acknowledges the complex interplay between socioeconomic factors and health outcomes, as demonstrated by Dahlgren and Whitehead’s Social Model of Health [15]. For example, this population often faces challenges in adopting and benefiting from health-promoting initiatives, as they are generally less likely to participate in such interventions [16, 17] and are more prone to drop out [18]. This socioeconomic gap in programme outreach and effectiveness might be due to the limited access to healthcare and resources, as well as reduced social support, all of which can significantly impact the overall well-being of this vulnerable demographic [19, 20]. Thus, besides addressing the complexity of healthy ageing and promoting overall well-being, an integral approach could potentially cater to the multifaceted and complex needs of older adults with low SES.

In addition to an integral approach, low-SES older adults could benefit from a systemic approach to healthy ageing. While both approaches promote well-being through comprehensive and integrated strategies, the systemic approach emphasises interaction and collaboration within systems such as policies, institutions and community practices and how this impacts overall health outcomes [21]. This procedure is closely aligned with the World Health Organisation’s (WHO) concept of healthy ageing, which underscores the necessity of reorienting health and social services towards person-centred and coordinated care models to support older adults’ functional ability and well-being [22]. Taken together, the active involvement of community-based support services such as home health care, social services, community centres, and the recreational sector plays a crucial role in creating an environment that supports healthy ageing of the elderly population.

Community involvement is another fundamental element for effectively promoting healthy ageing among low-SES older adults, as it enhances health outcomes and behaviours among disadvantaged populations [23]. This is a logical consequence since engaging communities and local neighbourhoods ensures that health initiatives and actions are more attuned to the unique needs and circumstances of low-SES individuals [23, 24], potentially fostering a feeling of ownership and collaboration among all residents and stakeholders involved [25]. Nevertheless, more research on integral and systemic community-based approaches is needed to inform local policies and practices about holistic strategies to support and enhance well-being [26]. For this purpose, we developed Neighbourhood Active & Connected (NAC; Wijk Actief in Dutch), a neighbourhood-oriented, integral and systemic approach to support healthy ageing in community-dwelling older adults with a predominantly low SES. In the context of this paper, the term *neighbourhood* refers to the home neighbourhoods of older adults, meaning distinct villages in rural areas and city districts in larger cities.

### A solid foundation for healthy ageing: Active Plus

During the preliminary stage of NAC’s development, we explored customising an existing intervention aimed at improving physical activity in older adults. The main reason was that sufficient physical activity is vital for healthy ageing, substantially benefiting our physical, emotional and social well-being. For instance, it improves general physical functioning [27, 28] and life expectancy [29, 30] and lowers the risk of developing chronic diseases [31–35]. Additionally, physical activity promotes positive mental health outcomes and acts as a protective factor against anxiety and depression in older adults [36, 37]. Moreover, through social settings like team sports, physical activity fosters social connections and a sense of belonging among older adults, combating loneliness and social exclusion, thus promoting overall well-being [38, 39].

**Fig. 1.**
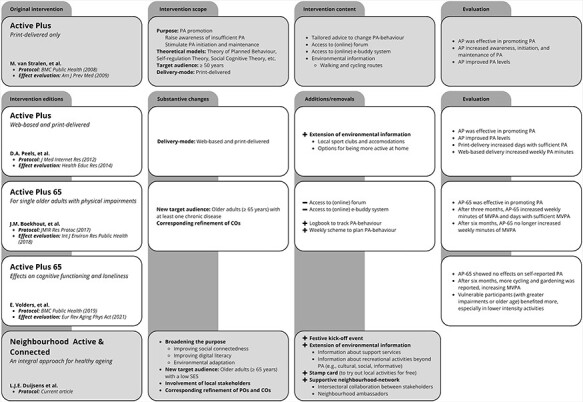
Evolution of the active plus (AP) intervention.

Therefore, the existing Active Plus (AP) intervention, designed to increase physical activity and social interaction in adults aged 50 and older, served as the foundation for the NAC intervention [40, 41]. The AP intervention is computer-tailored, as it uses a computerised system to produce three personalised letters of advice (digital or written) for the participants, customised to their unique qualities and interests. The intervention involves collecting data through web-based or written questionnaires, which serve as a data source for generating tailored messages that are selected based on decision rules that match the needs of each participant [40, 41]. Previously, the AP intervention yielded significant improvements in physical activity levels of adults aged 50 and over [42, 43]. Thereafter, AP was optimised for an older and vulnerable target group (AP-65) [44], after which it was able to promote physical activity [45] and reduce loneliness among single, chronically impaired older adults [46]. To clarify this timeline, Fig. 1 provides an overview of AP’s evolution, illustrating the successive editions of the AP intervention.

Despite its promising aspects, a recent study by Volders and colleagues revealed that AP-65 had no significant effect on self-reported physical activity among older adults with chronic diseases [47]. Nevertheless, the most vulnerable participants, especially those with greater physical impairment or older age, appeared to benefit more from the lower-intensity activities provided by AP-65. Although AP and AP-65 had positive outcomes over time, research indicated the necessity to investigate further ways to tailor the intervention better to address the needs of vulnerable older adults [47]. Additionally, a suggestion was made to transform AP into a blended approach by integrating add-ons aimed at personal interactions, professional support or environmental networks. Consequently, the NAC intervention builds upon AP and AP-65, meaning it will neither be a new stand-alone intervention nor an adaptation of AP’s existing components. Instead, the current study expands the original intervention with a broad shell of systemic (support) strategies to broaden its initial scope.

Accordingly, this paper aims to describe NAC’s development process using the Intervention Mapping (IM) framework to transform an evidence-based physical activity intervention into a holistic approach for healthy ageing. This involves detailing the steps taken to broaden the scope of the original AP intervention to better address the multifaceted needs of low-SES older adults. By outlining both the theoretical framework and the practical steps undertaken, this paper also aims to provide a detailed roadmap for researchers and practitioners looking to adapt and implement similar interventions in their communities, ultimately contributing to the body of knowledge on promoting healthy ageing in low socio-economic contexts. Consistent with the objectives of this paper, a separate Results section is not included. Instead, the Methods section thoroughly describes the steps of the IM framework, its application in NAC’s development, the intervention content and the decisions made regarding implementation and evaluation procedures. The Discussion section reflects on this entire process.

## Methods

In developing the NAC intervention, the IM framework was a guiding protocol, offering a standardised procedure for designing, implementing and evaluating health-promoting programmes [48]. Comprising six steps, IM begins with identifying the target population’s needs and results in proposing potential solutions. While typically used for designing new interventions, IM is also generally applied to adapt existing ones.

Furthermore, NAC’s development involved co-creation with older adults (≥65 years) with predominantly low SES and local stakeholders to tailor the intervention optimally for the target audience. This study defined local stakeholders as professionals and volunteers who regularly work with older adults, such as physical therapists and social workers, and parties involved in recreational activities, such as elderly associations and community centres. The methods section outlines the six steps of the IM framework and how they have been applied to develop NAC, in which the intricacies of how co-creation was employed throughout the process will be discussed more extensively.

### Study area

Before commencing the IM roadmap, specific geographical areas were selected as focal points for developing the NAC intervention and tailoring it towards existing assets and resources. This decision was made due to the intervention’s neighbourhood-oriented focus, ensuring that it could be customised to suit distinct characteristics and requirements of different communities.

Five municipalities in the south-east of the Netherlands volunteered to participate in the current study: Bergeijk, Bladel, Eindhoven, Heerlen and Voerendaal. This varied group, comprising three rural villages (500–1000 residents/km^2^) and two urban cities (≥1500 residents/km^2^), provided a good mix in terms of urbanisation, allowing for the investigation of potential links between city life and healthy ageing. After evaluating demographics (e.g. age composition of the population), socioeconomic characteristics (e.g. educational level and household income) and health indicators (e.g. being overweight and physical activity behaviour), each municipality selected two similar neighbourhoods or districts for participation in the current study. Within each municipality, these two selected neighbourhoods were randomly assigned to a specific role within the current study. The other neighbourhood ultimately acted as a control group during the intervention evaluation, which will be explained in further detail in Step 6. Municipal authorities, local elderly associations and special interest groups were the first to be informed about each neighbourhood’s assignment, as they played a pivotal role in engaging other stakeholders and fostering an integral support network at the neighbourhood level.

### Step 1: Needs assessment

The first step of the IM framework involves conducting a needs assessment and laying the groundwork for intervention development [48]. To fully understand what factors prevent older adults from ageing healthily, information was gathered on determinants that influence healthy ageing and their impact on the target group’s quality of life. This data was collected to provide background information on our target group, the context of healthy ageing, and the wider physical and social environment in which it manifests. By better understanding the context, the final intervention can be more effectively tailored to the target group’s specific characteristics, needs, resources and capacities. In the current study, the needs assessment consisted of consulting relevant literature and in-depth interviews. Its findings were subsequently (Step 2) translated into specific intervention goals for the NAC intervention.

#### Healthy ageing beyond physical activity

A holistic approach to promoting healthy ageing should encompass more than just physical activity, acknowledging its multifaceted nature and the numerous factors that influence well-being in older adults. This notion is likewise emphasised in the WHO’s definition of healthy ageing and in theories like *Positive Health*, both centred on improving quality of life by optimising opportunities related to physical and mental health, participation, daily functioning and finding a sense of purpose [11, 49]. Therefore, it became evident that the original AP intervention (as outlined in the Introduction and Fig. 1) needed to be broadened, as its objectives and scope were no longer entirely adequate. As a result, our initial aim in Step 1 was to identify additional relevant aspects of healthy ageing that could be integrated into the upcoming NAC intervention.

##### Social connectedness

Loneliness among older adults is a prevalent issue that can significantly impact their overall well-being. In the Netherlands, the prevalence of loneliness among older adults between 65 and 75 years was reported to be 34.0% in 2022 and continues to increase as individuals grow older [50]. These statistics are concerning, as loneliness is associated with increased healthcare consumption [51] and is considered to be a risk factor for depression [52] and mortality [53]. Thus, addressing loneliness and social isolation among older adults is vital for promoting healthy ageing and their overall well-being.

Interventions that target social engagement, peer support, and community activities have shown promise in mitigating loneliness and improving the quality of life for older adults [54, 55]. Promising components of these interventions included educational or supportive activities for older adults, encouraging their participation in decision-making, building community capacity, and involving community resources [55–57]. Such components would align well with an integral neighbourhood-oriented approach to healthy ageing, where it is essential to consider the local environment and its assets. Indeed, community-based interventions have effectively reduced loneliness by building a network between community assets and providing opportunities for older adults to interact with others [58]. By recognising and leveraging the strengths and resources within a neighbourhood or community, we can potentially enhance the effectiveness of interventions. Taken together, we decided to incorporate the strengthening of social connectedness and cohesion as a focal point for NAC, by considering the strengths and opportunities within the respective neighbourhoods, aiming to create a more supportive and inclusive environment for older adults.

##### Digital literacy

Technology-based solutions, such as computer and Internet usage, can significantly promote healthy ageing by offering various benefits to older adults. First of all, digital applications have been identified as tools that can contribute to older adults’ well-being by enhancing their interpersonal interactions and promoting cognitive functioning [59]. Second, Internet usage helps older adults compensate for their declining capabilities needed for daily activities and enhances autonomy [60]. Well-known practical examples of this are online grocery shopping, which enables older adults to have groceries delivered to their doorstep or the use of eHealth services to consult with healthcare professionals from the comfort of their homes.

However, older adults find it challenging to navigate the rapid digitalisation, everyday use of technological applications, and the increased usage of eHealth [61, 62]. Furthermore, this ‘*digital divide*’ exacerbates existing social health inequalities, predominantly instigated by limited material access to digital technologies and inadequate knowledge or skills [62, 63]. Based on this information, improving digital literacy was added as a crucial focal point in the NAC intervention. By focusing on digital skills development, we hope to empower older adults to fully participate in the digitising world and support their autonomy, ultimately promoting their overall well-being. The incorporation of social connectedness and digital literacy into the NAC intervention is further concretised in Step 2.

#### In-depth interviews

To further substantiate our needs assessment through co-creation, we conducted in-depth interviews. In total, 41 community-dwelling older adults were recruited, of whom 37 (aged 65 to 91 years) ultimately participated in the interviews. The majority of the interviewed older adults were female (62%), had lower levels of education (57%), and resided in rural areas (73%). Additionally, 71 local stakeholders working in sectors such as local government, primary and paramedical care, social services, leisure activities, and voluntary work were also involved. Recruitment employed the snowball sampling method, initiated by municipal authorities who identified potential stakeholders who had direct contact with older adults, who in turn presented potential candidates from their own professional networks. Likewise, these stakeholders facilitated connections with older adults residing in the neighbourhoods of interest, who were often consumers of their services. These older adults were then asked to establish contacts with acquaintances and neighbours who might be interested in participating, and so recruitment continued.

Data were collected through semi-structured interviews conducted from March to November 2021, meaning that this process primarily occurred during the COVID-19 pandemic. As a result, many interviews were conducted digitally (e.g. via Microsoft Teams), particularly with stakeholders, as this target group was more likely to be digitally literate. Conversations were held primarily one-on-one and occasionally involved companions, referring to a loved one among older adults or a colleague among stakeholders. The interviews generally lasted 45 to 60 minutes, covering physical activity, social connectedness, digital literacy, stakeholder collaboration, and strategies to engage older adults. Participants were asked to reflect on their needs, aspirations, and perceived (local) opportunities for improvement. All interviews were transcribed and thematically analysed.

All areas of concern and intervention recommendations derived from the interviews were rigorously evaluated for their feasibility and success potential. An example of a key theme that emerged during the interviews was the general lack of awareness regarding available community services and recreational resources. Many older adults reported not knowing what supportive resources existed within their neighbourhoods nor where to find relevant information. Parallel to these findings, interviews with local stakeholders revealed their concerns about the limited visibility of the local support network. They highlighted the need for a cohesive overview of active organisations, their respective roles and expertise, and the means to connect with them. Thus, the absence of a local *resource map* hindered not only older adults’ ability to access services but also stakeholders’ capacity to coordinate community support efficiently. In response to these findings, we re-evaluated the original AP intervention, which initially focused on sports providers registered with the Dutch industry association *NL Actief*. This initial emphasis on physical activities alone did not adequately address the diverse needs and preferences of the older population concerning healthy ageing. To address this gap, the intervention was revised to include a more extensive and localised directory of services, which was available both digitally and in a paper brochure. The revised version broadened the scope of activities beyond formal sport and exercise, incorporating various social and recreational activities, such as card games and community social events. Furthermore, information about supportive services such as district nursing was added, catering to the practical needs of older adults. In addition to this service directory, an additional intervention objective was incorporated into the NAC intervention, which sought to actively enhance participants’ knowledge of community resources and how to access them. The conceptualisation of this newly added performance objective will be elaborated upon in Step 2.

Table I provides an overview with additional examples of (sub)themes that emerged from the thematic analysis of our needs assessment [64, 65]. To supplement the interviews, group discussions were organised with local stakeholders and municipal authorities to evaluate our qualitative findings in detail. These sessions aimed to assess the practicality, resource availability, and alignment of the new intervention with existing programmes and initiatives in the neighbourhood. These collaborative efforts gathered feedback on feasibility, potential barriers, and necessary adjustments to inform decision-making. Subsequently, approved suggestions were carefully integrated into NAC’s intervention design, ensuring that they addressed the identified needs and aspirations and effectively leveraged local resources.

**Table I.** Examples of (sub)themes emerging from analysis of interviews during Step 1

Target group	Main theme	Subthemes	Remarks from participants
**Older adults**	**Physical activity**	Barriers to physical activity	Physical disabilities Disabilities of loved ones Failing to prioritise it Fear of getting injured
		Facilitators of physical activity	Social support and contact Professional coaching Accessible facilities
	**Digital literacy**	Resistance towards digitisation	Fast pace of implementation Feeling forced to become digitally literate Fear of scamming/phishing
		Motivation for becoming digitally literate	Maintaining independence Maintaining social contact Keeping up with society
	**Local activities and support services**	Threats to the local offer	(Too) limited offer Financial barriers Dissolution of clubs, associations, and facilities
		Recommendations for the local offer	Improved municipal support Improved affordability Involvement of older adults in decision-making
**Stakeholders**	**Socioeconomically disadvantaged older adults**	Barriers to providing assistance	Limited awareness of target audience Cultural norms and standards Social restraint
		Recommendations for providing assistance	Improving accessibility of services Integral approaches Raising awareness
	**Societal developments**	Societal concerns	Ageism Individualisation Social segregation
		Perceived role of the municipality	Facilitating collaborative efforts Financial support Training and education
	**Intersectoral collaboration**	Risks for collaboration	Privacy legislation Slow formation and progress Unwillingness
		Prerequisites for collaboration	Communication Environmental adaptation and sensitivity Independent leadership

### Step 2: Programme outcomes and objectives

The second step of IM involves specifying what behavioural and environmental changes should be made to achieve the desired intervention goals [48]. This is done by establishing a matrix of performance and change objectives. *Performance objectives* (POs) define the target group’s or environmental agents’ precise behaviours to attain the intended change. Combining these POs with specific determinants into so-called *change objectives* (COs) lays the groundwork for selecting theory- and evidence-based strategies and intervention components in Step 3.

#### Performance objectives

The original AP intervention consisted of eight POs focused on raising awareness and initiating or maintaining physical activity in older adults (Additional file 1; [44]). For NAC’s development process, these original POs were reconsidered, reevaluated, and complemented to align with the broadened focus of the NAC intervention, as described in Step 1.

The extension of the original AP intervention to an integral and systemic approach, coupled with findings from our needs assessment, led us to include two more additional intervention objectives for the NAC intervention. These additions included (a) raising awareness about local activities and support services and (b) fostering intersectoral cooperation among stakeholders. During Step 2, all aforementioned intervention objectives were translated into POs. The NAC intervention built on AP’s existing POs regarding physical activity by incorporating new POs to cover social connectedness, digital literacy, awareness of local activities and support services, and intersectoral collaboration between local stakeholders. Table II provides an overview of all new POs formulated for the NAC intervention.

**Table II.** POs for the NAC intervention

PO 1	(Continue to) meet the national exercise guidelines
PO 1.1 to PO 1.8	Original POs of Active Plus (Additional File 1)
PO 1.9	OA participate in local physical activity or exercise activities
PO 1.10	OA combine physical activity with local social activities
**PO 2**	**Strengthen social cohesion and (individual) social networks**
PO 2.1	OA develop new contacts and expand their social network
PO 2.2	OA are receptive for making new contacts
PO 2.3	OA participate in local social activities
**PO 3**	**Increase the use of digital devices and improving digital skills**
PO 3.1	OA adopt a positive attitude towards digital devices and digitisation
PO 3.2	OA specify purposes for which they want to use digital devices
PO 3.3	OA consult local support services for improving digital literacy
**PO 4**	**Improve awareness of local activities and support services**
PO 4.1	STK align their informational and supportive actions with the needs of OA
PO 4.2	STK actively inform OA about local activities and services that suit their needs
**PO 5**	**Enhance intersectoral collaboration surrounding OA**
PO 5.1	STK act from a shared perspective of supporting and encouraging healthy ageing
PO 5.2	STK involve relevant parties necessary to address or prevent problems in OA
PO 5.3	STK form an agreement on responsibilities and roles regarding healthy ageing in OA
PO 5.4	STK identify, support and refer OA to other relevant parties within the neighbourhood

PO Performance Objective, OA Older adults, STK Local stakeholders.

#### Change objectives

The following task involved identifying relevant determinants influencing the formulated POs and translating them into COs. It is important to note that this task was only carried out for newly established POS, as the original POs of AP (and AP-65) were already grounded in theory [40, 41], and therefore, its underlying COs remained unchanged. However, the categorisation of determinants for behavioural change in NAC’s matrix was derived from the original framework used for the AP intervention [44, 47] and supplemented by findings from the needs assessment (Step 1).

To illustrate this process, Table III depicts a small example from the established matrix of POs and COs devised for the NAC intervention. For instance, *knowledge* emerged as a relevant and modifiable determinant for POs 3.1 to 3.3 (Tables II and III). Regarding digital literacy, our needs assessment revealed that older adults often lacked knowledge about the capabilities of digital devices, the potential benefits of digitization and the available digital support services in their neighbourhood, hindering them from utilising online resources effectively [64]. To address this concern, COs were formulated to enhance older adults’ understanding of digital possibilities and local support services, including digital device functionalities and identifying opportunities for local support concerning digital skills.

**Table III.** Examples of COs for the NAC intervention

	*Determinants*			
Performance Objectives	Awareness	Knowledge	Skills and self-efficacy	Facilitation
PO.3.1. OA have a positive associations with digital devices and digitisation	A.3.1.a. OA recognise common misconceptions and (safety) concerns people have about using digital devices A.3.2.a. OA recognise the societal need to digitise certain sectors and processes	K.3.1.a. OA list the various benefits of using digital devices and engaging with digitisation K.3.1.b. OA recognise digital security risks and signs indicating phishing or fraud K.3.1.c. OA describe solutions for overcoming age-related challenges (e.g. vision or dexterity issues) regarding the usage of digital devices	SSE.3.1.a. OA express confidence in safely navigating the Internet and digital applications SSE.3.1.b. OA express confidence in overcoming age-related challenges (e.g. vision or dexterity issues) with adaptive technologies and tools	F.3.1.a. STK introduce OA to user-friendly digital devices and applications that are accessible and intuitive for their age group
PO.3.2. OA specify purposes for which they want to use digital devices	A.3.2.a. OA recognise specific situations or activities where digital devices can be helpful A.3.2.b. OA realise how incorporating digital devices can positively impact their daily activities and routines	K.3.2.a. OA describe the various functions and capabilities of digital devices K.3.2.b. OA list ways in which digital devices can be incorporated to enhance daily life: staying connected, accessing health resources, pursuing hobbies, etc. K.3.2.c. OA describe how using digital devices can contribute to their sense of independence and autonomy	SSE.3.2.a. OA express confidence in using digital devices to achieve their personal goals and aspirations	F.3.2.a. STK tell OA about means by which digital devices can be used in their daily lives, appropriate to their needs (e.g. online shopping, eHealth, managing personal finances)
PO.3.3. OA consult local support services for improving digital literacy	A.3.3.a. OA grasp the importance of developing basic digital literacy skills to effectively use digital devices A.3.3.b. OA acknowledge concerns and barriers they have about using digital devices	K.3.3.a. OA list the available resources and support services for digital assistance K.3.3.b. OA appoint the ways in which they can call for help, use or sign up for local support services	SSE.3.3.a. OA select local support services that match their personal needs, goals and interests regarding digital devices SSE.3.3.b. OA express confidence in seeking help from local support services	F.3.3.a. STK organise activities where OA can seek assistance, ask questions, and receive guidance regarding digital technology F.3.3.b. STK create a non-judgemental and supportive learning environment where OA feel comfortable asking questions, making mistakes, and seeking help F.3.3.c. STK offer resources such as tutorials, guides, and workshops tailored to the specific needs and interests of OA

OA Older Adults; STK stakeholders.

### Step 3: Programme design

The third step of the IM framework involved aligning theory- and evidence-based methods for behavioural change with the intended COs and developing practical applications to implement these methods in everyday practice. In this step, *theoretical methods* represent general approaches for altering determinants that influence behaviour or the environment, whereas *practical applications* are tactics to implement these methods and put them into practice [48].

#### Preserving the evidence-based foundation

For this paper, reviewing and optimising the underlying methods and applications of the original AP and AP-65 interventions was briefly considered. This review seemed pertinent given that AP and AP-65 were both fundamentally physical activity interventions, while NAC was designed to adopt a holistic and systemic approach. However, similar to Step 2, we retained AP’s original theoretical methods and practical applications due to compelling evidence of its efficacy in prior studies [45, 66]. It is crucial to highlight that modifying these foundational methods and applications could potentially undermine the proven-effective basis upon which the NAC intervention relies. Thus, instead of altering the core AP intervention, we supplemented it with additional methods and applications to address the newly introduced POs and COs unique to the NAC intervention, drawing from literature and a stakeholder survey, which will be elaborated upon in the following paragraph.

#### Incorporating behaviour change techniques from everyday practice

During Step 3, the local environment was called upon to further complement AP’s existing set of methodologies for NAC’s holistic and integral approach. Our conversations with older adults and local stakeholders during the needs assessment identified numerous existing initiatives supporting healthy ageing that were already in place, such as walking groups, coffee meet-ups, and consultation hours for questions related to digital activities. Hence, rather than introducing entirely new activities and methodologies, NAC’s development shifted towards improving older adults’ awareness of existing nearby opportunities for healthy and active ageing. These *nearby opportunities or initiatives* were defined as existing local activities, programmes, and services supporting healthy ageing. These initiatives encompassed a broad spectrum of community-based efforts intended to enhance the overall well-being of older adults. While key components included promoting physical activity, fostering social connectedness, and improving digital literacy, the initiatives involved in the NAC intervention were not restricted to these three domains. Other areas critical to the health and well-being of older adults, such as environmental conditions and housing support, were also considered.

First, all existing nearby opportunities and initiatives were mapped for each neighbourhood and evaluated to identify strengths, gaps, and their relevance to the NAC intervention’s objectives. Subsequently, to pinpoint behavioural change techniques already applied in local practices, 23 stakeholders across five Dutch municipalities were surveyed. This sample consisted of stakeholders active in the recreational sector, the (para)medical field, and social welfare. The survey contained 15 statements linked to behavioural change techniques as previously outlined by Kok and colleagues [67]. For each statement, local stakeholders could indicate whether or not they apply (similar) techniques or strategies in their daily practice (Additional File 2). The behaviour change techniques most frequently employed by local stakeholders were persuasive communication (95.7% of all cases), enhancing network linkages (91.3%), and creating opportunities for cultural similarity (73.9%). Techniques that stakeholders were least likely to use were discussion (34.8% of all cases) and self-monitoring of behaviour (26.1%), partly due to doubts about their practicality. Building on these findings, the NAC intervention sought methods to reinforce elements of the original AP intervention with the behavioural change techniques already in place and vice versa. Raising consciousness as a technique, for instance, was facilitated not only through AP’s computer-tailored advice letters but also through live seminars and workshops focusing on adopting a healthy lifestyle that were already in place. A complete overview of the survey results, the involved stakeholders, and the nearby initiatives they identified is provided in Additional File 3.

### Step 4: Programme production

The fourth step of the IM framework entailed preparing all necessary intervention messages, materials, and protocols [48]. Since NAC was built upon the AP intervention, adjustments were made to align AP’s existing protocols and materials with NAC’s newly formulated methodologies and applications. Materials from Boekhout’s sub-version of the AP intervention (AP-65) mainly formed the foundation, as this sub-version was optimised for participants aged 65 and over [44].

A significant challenge during this stage of NAC’s development was scaling up the original intervention into an integral and neighbourhood-oriented approach. Consequently, the intervention materials required revision from an entirely new angle. In addition to AP’s tailored physical activity advice, considering each participant’s unique living environment and local resources became paramount in the new NAC intervention. Moreover, an integral approach necessitates close collaboration and effective communication between local stakeholders in each neighbourhood, which is not always straightforward. This would entail NAC intervening in a completely new focus area and target group compared to the AP intervention. We engaged in extensive consultation and collaboration with older adults and local stakeholders to address these challenges. Suggestions from both target groups informed the revision and enhancement of AP’s materials, after which the updated intervention materials and protocols underwent evaluation in a small-scale pilot test.

#### New and adjusted components

First, the intervention was renamed Neighbourhood Active & Connected (‘Wijk Actief’ in Dutch) to reflect better its new neighbourhood-oriented approach and emphasis on social and digital connectedness. Consequently, all existing AP intervention materials underwent adjustments to align with the programme’s new identity, complete with a new corresponding logo. Furthermore, to improve accessibility for low-SES older adults, a Dutch linguist meticulously revised all questionnaires, ensuring they were comprehensible to Dutch speakers with language proficiency at level B1 according to the Common European Framework of Reference for Languages (CEFR) scale [68].

Subsequently, expanding on the AP intervention, information on close-to-home activities was further expanded and specified, now closely tailored to the neighbourhood instead of the regional or municipal level. By collaborating with local older adults and stakeholders, the planning team created detailed overviews of activities and support services available in each neighbourhood. This collaborative effort empowered local communities to actively contribute to and co-design the intervention, with particular attention to their unique social and physical environment.

Additionally, two so-called ambassadors were enlisted per neighbourhood to enhance community engagement and communication during the NAC intervention. These ambassadors were identified as local ‘key figures’ by municipal authorities, ranging from district nurses to board members of local elderly associations, and played a pivotal role in bridging the gap between scientific research, practical implementation, and the local community itself. These ambassadors’ installation resulted from the group discussions following the needs assessment, as described in Step 1. During these conversations, local stakeholders and municipal authorities suggested multiple times to involve local key figures, as it was believed that they could positively impact the acceptance of the NAC intervention by older adults in the community. Generally, people were thought to be more likely to participate when the intervention was presented and supported by a familiar face. This was reinforced by insights from the WHO, who identified numerous benefits of including target group representatives [69]. Thus, the involvement of neighbourhood ambassadors was deemed a crucial addition to the intervention, particularly given the perceived challenge of reaching vulnerable low-SES populations.

Next, since intersectoral collaboration formed the cornerstone of NAC’s strategy, a declaration of intent was introduced and signed by all stakeholders involved. Stakeholders from diverse backgrounds and expertise committed to acting from a holistic perspective, with the shared goal of promoting healthy ageing and enhancing the well-being of older adults within their communities. Specific examples of stakeholders involved were community centres, district nurses, GP practices, libraries, physical therapists, seniors’ associations, social workers, social welfare advisors, and sports facilities. In the declaration, stakeholders outlined their respective contributions to the partnership, fostering an environment favouring intersectoral collaboration and joint actions addressing (socioeconomic) health inequalities.

Last, the launch of the NAC intervention in each neighbourhood was marked by a celebratory kick-off event, providing a platform for local stakeholders to showcase their services tailored to older adults. This free-to-attend gathering facilitated personal interactions, allowing older adults to explore various activities and forge connections with stakeholders. Practical workshops and networking opportunities during the kick-off event further underscored the holistic nature of the intervention, showcasing how different aspects of healthy ageing can synergise to promote a healthier and supportive local community. A complete overview of the NAC intervention and its adjustments compared to AP is presented in Additional File 4.

#### Adaptation of delivery channels

Participants of the AP intervention could opt for an online (web-based) or offline (printed) intervention delivery. Online participants completed all questionnaires on the intervention website via email invitations, while offline participants received pencil-on-paper questionnaires by mail. The online version of the intervention was web-based and could, therefore, be accessed on laptops, tablets, and smartphones. This flexibility allowed participants to choose the device that best suits their needs and easily switch devices if necessary. The content of the tailored advice letters remained consistent across delivery modes, albeit with practical differences (e.g. the offline version featured images, while the online version included videos) [40, 44].

Despite increased Internet usage among older age groups, Dutch older adults and those with a low SES still utilise the Internet less frequently than younger and wealthier individuals [70]. Hence, to best accommodate NAC’s target population, both delivery modes were retained, allowing participants to select their preferred delivery method. Participants of the NAC intervention indicated their preference for the online or offline intervention delivery on the enrolment form, after which they received all intervention materials accordingly.

**Fig. 2.**
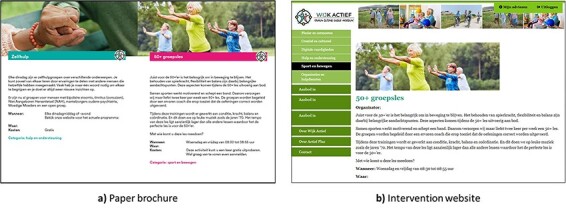
Local activities as presented in the paper brochure (a) and the intervention website (b).

As previously described, the NAC intervention provides a comprehensive overview of the local environment compared to AP. All participants received a newly formed paper brochure tailored to their neighbourhood, featuring local activities and a welcoming message from their municipality (Fig. 2a). The same content was also digitised and integrated into the intervention website for online participants (Fig. 2b). Given the holistic nature of NAC, stakeholders likewise had to be fully aware of local activities and support services. Therefore, unlike prior versions of the AP intervention, the NAC intervention website was made publicly accessible, allowing interested stakeholders and community members to freely access the environmental information without login credentials, even when they did not actively participate in the intervention.

#### Pilot test

Although the materials of the AP intervention had undergone prior evaluation, an additional pilot test of the NAC intervention and its materials was conducted. This small-scale pilot test (*N = *10) aimed to evaluate the usability and feasibility of the intervention through a mixed-methods approach, incorporating a short paper-based form and follow-up phone interviews. The form asked older adults to evaluate NAC’s information letter and questionnaires for usability, comprehensibility, and attractiveness. Generally, participants found the materials easy to read and understand, scoring them highly for comprehensibility (8.1 for the information letter and 8.0 for the questionnaires on a scale of 1–10). During the telephone interview, participants could explain their answers on the form by verbally expressing their opinions and experiences. For instance, some participants expressed concerns about the materials’ length, suggesting that overly lengthy documents could hinder participation.

In response, we collaborated with the pilot test participants to explore ways to shorten and simplify NAC’s intervention materials. One suggestion was to format the information letter graphically, using clear headings, colours, and images akin to an advertising pamphlet, facilitating quick comprehension while retaining all the necessary information. Despite the inability to shorten the questionnaires, due to the essential nature of some items for tailoring the physical activity advice letters, other adjustments were made to enhance the experience. For instance, the order of items was shuffled, and a progress bar was added to the online version to allow participants to track progress and pause as needed. Furthermore, a Dutch linguist converted the questionnaires to CEFR language level B1. Nine low-literacy adults reviewed these converted questionnaires again, resulting in minor textual changes based on their feedback.

### Step 5: Programme implementation

An effective intervention hinges on successful implementation, epitomising the main purpose of Step 5 within the IM framework. Here, a plan was established to facilitate the adoption and implementation of the NAC intervention [48]. The ultimate impact of interventions relies on both their effectiveness and their real-world application. Therefore, a systematic process is necessitated for creating an effective implementation strategy, which considers determinants of implementation, the selection of theoretical methods and strategies, and the evaluation of implementation results [71]. The implementation plan for the NAC intervention in everyday practice will be drawn from insights gained from a prior implementation study [72], our experiences during the intervention evaluation (as described in Step 6), and valuable perspectives obtained through interviews with stakeholders who may be involved in the future implementation of the NAC intervention.

A prior implementation study regarding the original AP intervention provided insight into the implementation outcomes of a multifaceted implementation intervention, which was developed according to the principles of the IM framework [48] and supplemented with relevant insights from the available literature on implementation science [73–76]. This initial study offered valuable insights into the use of implementation strategies, the achievement of POs, changes in implementation determinants, and implementation output that are relevant for the implementation of the NAC intervention as well.

In the prior study, feasibility results (reflected in using implementation strategies and achieving POs) demonstrated that stakeholders broadly accepted the developed implementation intervention. However, the implementation determinants did not change favourably, and stakeholders generally had no plans to continue the original AP intervention [72]. As the most important reason for not continuing the implementation, implementers declared the unexpected amount of effort needed to engage stakeholders and end-users as the primary reason for halting the implementation. Consequently, in the current project, efforts have been made to enhance other relevant stakeholders’ engagement and develop recruitment strategies that target the end-users.

Subsequently, to evaluate NAC, we established connections with five Dutch municipalities interested in adopting the forthcoming intervention, as mentioned before. Through concerted networking efforts and strategic lobbying, this partnership expanded further to include regional public health services and pertinent knowledge partners, including the Dutch National Centre of Expertise on Addressing Health Inequalities (PHAROS). To fortify local commitment and relevance, each municipality’s engagement was further reinforced through sustained dialogue with local councillors, policy advisors, district coordinators, and social workers, facilitated by our research team’s ongoing support.

A multi-channel communication strategy ensured widespread awareness of the upcoming intervention among the end-users. This was partially influenced by input from older adults gathered during the needs assessment (Step 1). The preferred communication channels of seniors varied widely, including paper flyers and direct mail, the local neighbourhood newspaper, Internet resources, and word-of-mouth communication. Based on this diverse feedback, we decided to employ a broad promotional strategy incorporating both analogue and digital approaches. At the start of the programme, formal invitations were mailed to all residents aged 65 and over based on municipal demographic data and address files, complemented by the support of local stakeholders to maximise information distribution. Additionally, various media outlets, such as television broadcasts, social media campaigns and advertisements in magazines and newspapers, were deliberately used to disseminate information to the elderly populace. This elaborative approach ensured optimal dissemination of information and prepared older adults for the impending intervention.

During NAC’s programme evaluation, we will consider the same implementation output measures as those used in our previous study on the AP intervention [48]. Implementation output will be assessed as the adoption rate (i.e. the number of municipalities agreeing to adopt the intervention divided by those invited), the reach of end-users (i.e. the number of people using the intervention divided by those invited), intervention drop-out rates among users (i.e. the number of people dropping out divided by the number that adopted the intervention), and implementation continuation among municipal healthcare policy advisors (i.e. the number of advisors choosing to continue implementation divided by the number that started implementation). When proven effective, additional interviews will be conducted with other stakeholders potentially involved in the future implementation of the NAC intervention to gather valuable insights into relevant implementation determinants. Following these interviews, matching implementation strategies will be discussed with the stakeholders as well, after which the intervention will be optimised for nationwide implementation.

### Step 6: Programme evaluation

The sixth step of the IM framework involved outlining a protocol for assessing the efficacy of the intervention and, subsequently, its evaluation. Despite being informed by evidence-based theories and methodologies, the success of an intervention is not guaranteed immediately. Therefore, a process evaluation is crucial for determining the efficacy of an intervention design, as it helps to measure the actual impact of the intervention and the decisions made during its planning stages [48]. A process evaluation assesses whether an intervention has achieved its behavioural, environmental, and implementation outcomes, and tries to understand the reasons behind them. This practice ensures consistent quality of the intervention, validates the ongoing resource allocation, and ultimately establishes responsibility in our health-promoting endeavours [48].

**Fig. 3.**
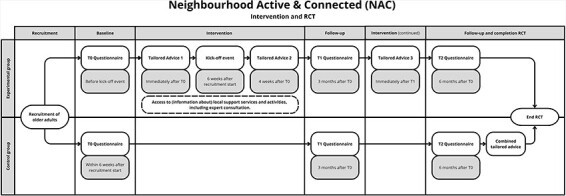
A graphic representation of the NAC intervention and its randomised controlled trial.

Thus, the final step of the present study involved formulating a protocol for a cluster randomised controlled trial (RCT) to evaluate the effectiveness of the NAC intervention. During this cluster RCT, older adults in experimental neighbourhoods (receiving the NAC intervention) will be compared to older adults in demographically matched control neighbourhoods within the same municipality. As previously described, all neighbourhoods were randomly assigned to one of both study arms, as outlined in Fig. 3. Ethical approval for the cluster RCT (reference number: U202209546) was authorised by the central ethical review committee of the Open Universiteit (Heerlen, the Netherlands), after which practical implementation of the cluster RCT lasted from February to December 2023. Additionally, the cluster RCT was registered at the ISRCTN trial registry (reference number: ISRCTN17170098). All participants provided written informed consent to participate in the study.

#### Participants

Community-dwelling older adults (≥ 65 years) were eligible for participation, excluding individuals diagnosed with cognitive impairment, who had insufficient Dutch language proficiency, or who could not independently access local facilities (e.g. community centres and supermarkets). Each participant was assigned to the experimental or control group according to their place of residence. Accordingly, participants were categorised as urban or rural residents, considering the potential impact of urbanisation on their health and well-being. In the Netherlands, urban areas generally report higher rates of loneliness, unhappiness, life dissatisfaction, and mental health issues [77–79]. Nevertheless, these individuals generally benefit from better healthcare accessibility and cultural amenities than their rural counterparts [80]. Hence, understanding intervention effects associated with urbanisation is crucial for a nuanced comprehension of the intervention’s impact.

#### Design and procedure

Six months before recruitment for the cluster RCT, local stakeholders who pledged their commitment to the NAC intervention underwent several training and informative sessions. In the meantime, each municipality commenced participant recruitment by sending information letters and application forms to all older adults in the designated neighbourhoods. The application form included the option to choose the online or offline intervention and could be directly returned to the research team. Afterwards, older adults opting for the offline intervention received a mailed package with the pen-on-paper questionnaire, an informed consent form, a return envelope, and an invitation to the kick-off event. Those choosing the online intervention received login details and all additional information via email.

The NAC intervention formally began after the completion of the baseline questionnaire (T0). The kick-off event, held approximately 6 weeks after the beginning of the recruitment period, provided the attending participants with a brochure containing environmental information. For those unable to attend, these materials were sent by post at a later time. Follow-up questionnaires occurred 3 (T1) and 6 (T2) months post-enrolment. The experimental group received three tailored advice letters between the questionnaires, while the (waitlist) control group received one elaborate tailored advice after completing the final questionnaire.

The cluster RCT did not include formal stopping criteria, for the NAC intervention was non-invasive and primarily targeted behavioural and social outcomes. Hence, the risk of adverse events or negative health outcomes was deemed minimal by us researchers and the ethical review committee. Moreover, as our cluster RCT focused on evaluating the efficacy of the NAC intervention, this aspect could not be predefined as a stopping criterion. Additionally, logistical challenges were anticipated as potential real-world obstacles, providing valuable insights for the future practical implementation of the intervention in everyday settings. Therefore, rather than establishing stopping criteria, the cluster RCT emphasised close monitoring to address any unforeseen issues promptly.

#### Measurements

The cluster RCT had four primary outcome measures: physical activity behaviour, loneliness, social cohesion, and health-related quality of life (HRQOL). Physical activity was assessed using the validated ‘Short questionnaire to assess health-enhancing physical activity’ (SQUASH), which was selected for its reasonable reliability and validity compared to accelerometry [81]. Furthermore, subjective measures like the SQUASH effectively differentiate between various activities (e.g. cycling, gardening, and household activities) and, thereby, also capture low-intensity physical activities prevalent among older adults [47]. Loneliness was measured with the De Jong Gierveld 6-item Loneliness scale, known for its satisfactory psychometric properties [82]. Social cohesion was assessed using the 6-item scale from the Dutch CBS Safety monitor (‘Veiligheidsmonitor’ in Dutch) [83]. HRQOL was quantified using the EQ-5D-5L questionnaire, which is widely recognised in clinical trials and observational studies [84, 85]. Secondary outcome measures included digital activity and literacy, evaluated through a shortened version of the Dutch CBS questionnaire on digital skills and Internet usage (‘ICT-gebruik huishoudens en personen’ in Dutch) [86].

Furthermore, participants provided demographics and information on factors influencing their physical activity behaviour, such as attitude, self-efficacy and perceived social support. In the experimental group, additional questionnaire items assessed their experience with the NAC intervention regarding ease of use, satisfaction, appeal, personal significance and comprehension. Logging data were collected throughout the intervention for participants using NAC’s web-based components.

Given the hierarchical structure of the data, with measurements nested within participants and participants nested within neighbourhoods, multilevel linear regression analyses were employed. This approach allowed us to account for the potential clustering at the neighbourhood level, ensuring that the cluster RCT’s findings accurately reflect the intervention’s impact while controlling for intra-class correlation (ICC).

Lastly, the intervention was evaluated after the last measurement through one-on-one interviews with older adults and local stakeholders. Evaluation criteria included the perceived image of the NAC intervention in the neighbourhood, satisfaction with the collaborative approach, establishment of local networks, and community outreach. Notes from these interviews and field observations complemented the overall evaluation process.

#### Power calculation

Our sample size calculation was derived from prior research on the AP intervention, which indicated a 0.4 effect size after 6 months [45]. It was assumed that similar effects would be observed in older adults with low SES. Anticipating effects on social cohesion, loneliness, and HRQOL, we expect an effect size of 0.35. To accommodate the multilevel design of our cluster RCT, adjustments to the standard sample size are necessary to consider the estimated ICC. Based on prior AP trials, the ICC is estimated at 0.035. With an anticipated effect size of 0.35, power of 0.80, alpha of 0.05, and ICC of 0.035, approximately 360 participants were required. Accounting for an expected 40% dropout rate during the cluster RCT, 600 participants at baseline were deemed necessary. Distributing this number across the five municipalities involved, approximately 180 participants per city and 80 participants per village were needed.

## Discussion

This paper aimed to describe the development process of the NAC intervention, a holistic and neighbourhood-oriented approach designed to promote healthy ageing among low-SES older adults. Leveraging the successful AP and AP-65 physical activity interventions [42, 45, 47, 87], NAC addresses the broader, multifaceted needs of low-SES older adults by incorporating elements such as professional support and information about local support services and resources. The NAC intervention was developed using the IM framework, ensuring a theoretically grounded and evidence-based approach [48]. This comprehensive method ensures that the intervention is closely tailored to the specific needs and circumstances of low-SES older adults, thereby enhancing its potential for success and contributing to creating holistic strategies for promoting healthy ageing in low-SES environments.

The newly developed NAC intervention introduces several strengths, making it a promising approach for promoting healthy ageing in older adults. First, it builds upon the proven effectiveness of the AP (and AP-65) physical activity intervention, ensuring a solid groundwork. Second, NAC offers online and offline components, accommodating diverse preferences and digital literacy levels. Third, the intervention’s co-creation process safeguards inclusivity and improves responsiveness to low-SES older adults’ actual needs and perspectives. Fourth, NAC is finely tuned to the specific context of each neighbourhood, acknowledging the importance of tailoring interventions to local nuances, such as the accessibility of resources and support networks. Lastly, a large-scale cluster RCT is planned to provide robust evidence of NAC’s impact on the health and well-being of older adults, collectively positioning it as a comprehensive and well-rounded intervention.

Many researchers and policy developers have recognised the importance of a holistic approach that transcends the focus on singular health aspects, advocating for interventions that incorporate physical, psychological, social, and environmental determinants of health. This paradigm shift is evident in recently developed interventions that address multiple life domains, such as the ‘Community Wise’ programme, which targets physical fitness, self-management abilities, social health, and well-being in low-SES older adults [88]. However, to our knowledge, the NAC intervention represents a pioneering effort by integrating this holistic framework with a systemic approach by actively involving local stakeholders in a collaborative response. Furthermore, a notable aspect of the NAC intervention is the inclusion of stakeholders outside the traditional realms of primary and social care [89]. By engaging diverse community-based support services such as community centres, libraries, sports clubs, and elderly associations, the NAC intervention leverages a broad network of resources and expertise. This interdisciplinary collaboration enhances the intervention’s capacity to comprehensively address the multifaceted needs of low-SES older adults.

Despite its strengths, the NAC intervention has several limitations that warrant acknowledgement. Foremost among these is the timing of its development and evaluation (September 2020 to December 2023), which coincided with the COVID-19 pandemic. This period brought unprecedented disruptions, including extensive lockdowns and strict social distancing measures, posing significant challenges to the integral approach’s design and implementation. Recruiting participants, particularly during the needs assessment phase, proved practically impossible, limiting access to a diverse and representative sample. Furthermore, data collected during the pandemic may only partially reflect the *normal* situation. Indeed, the very foundation of communal life was radically disrupted, which halted many neighbourhood activities and initiatives for older adults. This hindered our efforts to establish meaningful connections with older adults and stakeholders—an essential aspect of our co-creation process.

Another significant challenge in our study is the reliance of NAC’s success on effectively engaging low-SES older adults. This demographic is often considered a hard-to-reach target group, necessitating the involvement of local stakeholders to optimise outreach. Additionally, the NAC intervention’s reliance on written information may pose difficulties for older adults with limited (health) literacy. We therefore hope that the partial conversion to CEFR language level B1 and the use of videos (online) or images (offline) would enhance the intervention’s accessibility.

Finally, applying the IM framework offers benefits and drawbacks that warrant evaluation. On the one hand, the framework proved well suited for developing an integral neighbourhood-oriented approach, considering individual, community and societal factors in line with the intended holistic perspective [48, 67]. Moreover, the IM framework prioritised the active involvement of local stakeholders throughout its process, aiding in tailoring the intervention to the unique needs of our vulnerable target group and their environment [48, 90]. However, applying the IM framework is inherently complex and time-consuming. In this case, the needs assessment alone took 11 months to complete, partly due to the disruptions caused by the COVID-19 pandemic. Therefore, executing IM’s systematic approach with limited time and resources may pose challenges, particularly in community settings.

To summarise, the NAC intervention is an integral neighbourhood-oriented approach to support healthy ageing in older adults with a low SES. The intervention appears to be a promising strategy for targeting numerous ageing-related risk factors while simultaneously facilitating the existing procedures and structures in community care for older adults. Before the intervention can be widely implemented, the effectiveness of NAC will be evaluated in a large-scale cluster RCT, of which the results will be published in future papers. Insights of our research will provide recommendations and implementation guidelines for regional public health services and municipalities planning to employ an integral and community-based approach targeting healthy ageing. When proven effective, the NAC intervention will be put forward to national public health agencies that will actively distribute the intervention and make it accessible to a broader audience (e.g. the general public and practice organisations).

## Supplementary Material

cyae041_Supp
