# Supplementary material for: A Neighbourhood-oriented approach to foster healthy ageing in low socioeconomic older adults: development and protocol for evaluation through intervention mapping
Source: Health Educ Res. 2024 Dec 14;40(1):cyae041. doi: 10.1093/her/cyae041 (PMC11840751; doi:10.1093/her/cyae041)
Supplement: cyae041_Supp [file cyae041_supp.zip › Supp_data/Additional_file_3.pdf]

	Neighbourhood 1<br>*Rural*	Neighbourhood 2<br>*Rural*	Neighbourhood 3<br>*Rural*	Neighbourhood 4<br>*Urban*	Neighbourhood 5<br>*Rural*
**Stakeholders surveyed**	• District nurses (N=2)<br>• Independent activity initiator (N=1)<br>• Municipal policy officers (N=1)<br>• Representatives of seniors association (N=2)	• Librarian (N=1)<br>• Representatives of interest groups (N=2)<br>• Representative of seniors association (N=1)	• Community centre (N=1)<br>• District nurse (N=1)<br>• Municipal policy officers (N=1)<br>• Neighbourhood support officer (N=1)<br>• Personal trainer (N=1)	• Independent activity initiator (N=1)<br>• Municipal policy officer (N=1)<br>• Personal trainer (N=1)	• Community centre (N=1)<br>• District nurse (N=1)<br>• Independent activity initiator (N=1)<br>• Municipal policy officer (N=1)<br>• Represenative of seniors association (N=1)
**Identified neighbourhood initiatives**	**Initiatives related to physical activity:**<br>• Bowls<br>• Cycling group<br>• Dancing class<br>• Fitness park<br>• Gymnastics group<br>• Petanque<br>• Pilates<br>• Tai Chi<br>• Walking group<br>• Walking trail with instructional tiles<br>• Water aerobics<br>• Yoga<br><br>**Initiatives related to social connectedness:**<br>• Bingo<br>• Billiards<br>• Card games<br>• Coffee meet-ups<br>• Day-care<br>• Knitting club<br>• Singing choir<br><br>**Initiatives related to digital literacy:**<br>• Theme-based workshops<br>• Training courses<br><br>**Other support services:**<br>• Assistance with tax returns and financial questions<br>• Consultation hours - District nursing<br>• Consultation hours - Municipal support<br>• Handyman service<br>• Informal caregivers support group<br>• Transportation service	**Initiatives related to physical activity:**<br>• Bowls<br>• Dancing class<br>• Gymnastics group<br>• Petanque<br>• Ping-pong<br>• Tai Chi<br>• Walking group<br>• Yoga<br><br>**Initiatives related to social connectedness:**<br>• Bingo<br>• Card games<br>• Coffee meet-ups<br>• Communal dinners<br>• Singing choir<br>• Women's advocacy association<br><br>**Initiatives related to digital literacy:**<br>• Consultation hours - Digital questions<br><br>**Other support services:**<br>• Consultation hours - Municipal support<br>• Grocery delivery<br>• Transportation service	**Initiatives related to physical activity:**<br>• Bowls<br>• Dancing class<br>• Gymnastics group<br>• Petanque<br>• Walking group<br>• Water aerobics<br>• Yoga<br><br>**Initiatives related to social connectedness:**<br>• Billiards<br>• Card games<br>• Communal lunch and dinners<br>• Crafting group<br>• Day-care<br>• Coffee meet-ups<br>• Handicraft and recycling group<br>• Knitting club<br>• Sewing class<br>• Singing choir<br><br>**Initiatives related to digital literacy:**<br>• Consultation hours - Digital questions<br>• Theme-based workshops<br>• Training courses<br><br>**Other support services:**<br>• Consultation hours - Informal caregivers<br>• Consultation hours - Municipal support<br>• Self-help groups<br>• Theme-based meetings on care and prevention<br>• Volunteer centre	**Initiatives related to physical activity:**<br>• Bowls<br>• Dancing class<br>• Golf<br>• Gymnastics group<br>• Petanque<br>• Shuffleboard<br>• Tai Chi<br>• Walking group<br>• Walking trails<br>• Yoga<br><br>**Initiatives related to social connectedness:**<br>• Bingo<br>• Billiards<br>• Card games<br>• Communal lunch and dinners<br>• Darts<br><br>**Other support services:**<br>• Consultation hours - District security<br>• Consultation hours - Municipal support	**Initiatives related to physical activity:**<br>• Cycling group<br>• Fitness park<br>• Geriatric physical therapy<br>• Gymnastics group<br>• Tai Chi<br>• Yoga<br><br>**Initiatives related to social connectedness:**<br>• Bingo<br>• Card games<br>• Coffee meet-ups<br>• Communal breakfast<br>• Community meetings<br><br>**Initiatives related to digital literacy:**<br>• Consultation hours - Digital questions<br>• Training courses<br><br>**Other support services:**<br>• Home visits for welfare and care related issues<br>• Online community platform
**Behavioural change techniques in place**					
**Conciousness raising**	Yes	No	Yes	Yes	Yes
**Cultural similarity**	Yes	Yes	Yes	Yes	Yes
**Discussion**	Yes	Yes	Yes	Yes	Yes
**Enhancing network linkages**	Yes	Yes	Yes	Yes	Yes
**Facilitation**	Yes	Yes	Yes	Yes	Yes
**Feedback**	Yes	No	Yes	No	Yes
**Goal setting**	Yes	No	Yes	Yes	Yes
**Guided practice**	Yes	Yes	Yes	Yes	Yes
**Individualisation**	Yes	No	Yes	Yes	Yes
**Participation**	Yes	Yes	Yes	No	Yes
**Persuasive communication**	Yes	Yes	Yes	Yes	Yes
**Planning coping responses**	Yes	No	Yes	Yes	Yes
**Public commitment**	Yes	No	Yes	Yes	Yes
**Self-monitoring**	Yes	No	Yes	No	Yes
**Social comparison**	Yes	No	Yes	No	Yes

Yes = At least one respondent used this technique; No = No respondent used this technique.
